# Species Extinction, Infrastructure Development and Epidemics: The Changing Ecology of African Horsesickness in the Cape Colony, *c.*1653–1900

**DOI:** 10.1080/03057070.2024.2508570

**Published:** 2025-07-15

**Authors:** Chris Andreas

**Affiliations:** University of Vechta

**Keywords:** African horsesickness, Cape Colony, epizootics, horse use, quagga extinction, *Culicoides* midges, water infrastructure, environmental change

## Abstract

The virus that causes African horsesickness does not affect any indigenous species, but produces high mortality among horses, a species introduced by the Dutch East India Company in 1653. While the insect-borne disease did not occur in the immediate vicinity of the Cape Peninsula, horsesickness could have constituted an endemic disease barrier to the horse-based expansion of the colonial sphere into the hinterland, where it was seasonally prevalent. That it did so to only a limited extent is due to a substantial alteration of the ecology of the disease that largely resulted from inadvertent side effects of anthropogenic modifications of the environment concomitant to the socio-economic development of the colony. This epidemiological transition evolved in two phases that overlapped chronologically but were clearly distinct regionally. It had started in the south-west of the Cape Colony in the later part of the 18th century and, in correlation with, first, the progressive extinction of quagga and zebra populations and, second, the economic intensification of pastoral and agricultural production and accelerated horse travel, gradually shifted over the following century toward its northern and eastern boundaries. During the earlier phase of this process, the area in which horsesickness was seasonally prevalent contracted steadily. However, the subsequent intensification of the utilisation of horses in transport and farming facilitated the recurrence of ever more frequent and economically devastating large-scale epidemics of horsesickness. The history of African horsesickness in the Cape thus provides an instructive example illustrating the unexpected consequences of human modifications of the environment.

## Introduction

Horses were a vital part of colonisation at the Cape. Introduced by the Dutch East India Company (VOC) in 1653, a year after the settlement was founded, they proved valuable for military purposes and for many forms of transport. They were also adopted by African people from the 18th century.^1^ But horses were assailed in much of Africa by a viral disease, African horsesickness, that caused widespread deaths.^2^ In most of the coastal and tropical areas of Africa, where it was regionally supplemented by trypanosomiasis, the disease proved so virulent that it was not feasible to sustain horses. The area that became South Africa was on the margins of this zone, as were some other drier, higher environments. This article explores how and why horsesickness changed in incidence and severity over the first two-and-a-half centuries of colonisation, even though it never fully undermined the centrality of horses in colonial life. The analysis, it is argued, requires understanding of the transmission of the disease, the ecology of the country, as well as the impact of colonial intrusion. Historical outcomes were shaped by the interaction of socio-economic, environmental and biological processes.

Southern Africa was home to wild equine species, Cape Mountain zebra (*Equus zebra*) and two subspecies of plains zebra (*Equus quagga burchelli*), previously known as Burchell’s zebra, and the now extinct, less conspicuously striped quagga (*Equus quagga quagga*).^3^ They, and especially the first and most widespread plains zebra, were likely reservoir hosts for this viral disease. Horsesickness does not affect any of the indigenous equines but produces high mortality – from at least 50 per cent up to 95 per cent – among domestic horses. It is transmitted by certain species of *Culicoides* midges. These are blood-sucking insects with highly specific moisture and temperature-dependent breeding requirements, that significantly determine the ecology of the disease. However, transmission by an insect was suspected only after 1900, and *Culicoides* were only identified as vectors as late as 1944.^4^ Because of the ecological conditions required by the midges, horsesickness affects horses seasonally only in some areas of southern Africa, but periodically sparks epizootic outbreaks during which it spreads over a much larger region.^5^

This article will demonstrate that within this general pattern – which scientists trace mainly to seasonal and multi-annual climate cycles – there was a marked and enduring transition in the ecology of horsesickness during the 18th and 19th centuries. This epidemiological transition evolved in two phases that overlapped chronologically but were clearly distinct regionally. During the first century of colonisation, reports suggest that this was a seasonal disease affecting most of the colony. From the late 18th century, the area in which horsesickness occurred seasonally contracted steadily from the south-western Cape toward the northern and eastern boundaries. But by the mid 19th century, the scale and frequency of economically devastating epizootics greatly increased. Epizootics were not annual nor predictable and swept rapidly through much of the Cape. During the early 20th century, their impact gradually diminished because of motorisation and the availability of a vaccine from 1934 onward. It will be argued here that besides climatic factors, anthropogenic modification of the environment caused by colonial development, in particular hunting and advances in water and traffic infrastructure, played an important role in this epidemiological transition of horsesickness. The phased nature of this transition, and some of its anthropogenic causation, has not yet been fully appreciated in the scientific literature, even though a key study explicitly adopts a historical perspective.^6^

There is a substantial historiography on animal diseases and the development of veterinary science in southern Africa that focuses predominately on zoonoses like trypanosomiasis and rabies, and epizootic cattle disease, especially rinderpest and East Coast fever, as well as some work on sheep and dogs.^7^ Much of the historiography looks at the veterinary and regulatory management of such diseases and the production of knowledge underlying it, particularly at scientific debates and discoveries in the period after government veterinary services were established from the 1870s.^8^ Environmental histories of the diseases themselves, as well as work on veterinary interventions during earlier periods, or work by Africans, are more rare.^9^

The small extant historiography of horsesickness similarly focuses on its scientific exploration from the establishment of public veterinary services in the late 19th century onward.^10^ For the earlier period, the disease inevitably features in histories of the horse, but an environmental history of horsesickness in the Cape that goes back into the 18th century is lacking.^11^ This article seeks to provide an exploratory survey that embeds this history into present scientific understanding of the disease and, perhaps, supports scientific research with a more detailed historical background.

## The Nature of the Horsesickness Problem in the Cape

African horsesickness is currently not endemic in South Africa, except for the north-eastern Lowveld in today’s Mpumalanga Province.^12^ The disease appears there each year in December or January and then spreads southward to an extent that is largely determined by the timing of its first incidence and the suitability of climatic conditions for the breeding of the *Culicoides* midges that transmit it. In the summer rainfall areas it is most prevalent in warm coastal regions as well as moist, low-lying inland areas like valleys and marshes, and along rivers. It is most virulent during the second half of summer, in March and April, and disappears abruptly after the first frosts in late April or May terminate vector activity.^13^

At present, the Highveld, the Free State and most of the Cape are free from such seasonal horsesickness incidence; only the coastal Transkei in the Eastern Cape is susceptible. In the past, the area affected by horsesickness every year was much larger, and it has been claimed that the virus used to be ‘endemic across virtually the whole of South Africa and outbreaks of AHS [African horse sickness] … are recorded on an almost countrywide basis throughout the whole of the 19th Century and into the first few decades of the 20th Century’. Somewhat imprecisely, this claim lumps together genuine endemic prevalence, annual seasonal incidence and sporadic large-scale epizootics.^14^ Yet the area of seasonal incidence as well as the frequency of such epizootics have varied greatly over the past centuries. Understanding the changes in the ecology of the disease and the problems it posed therefore requires a more differentiated historical analysis.

### Seasonal Horsesickness

While seasonal horsesickness was endemic to the more tropical summer rainfall area of South Africa, it was not just a ‘scourge of the wet season’;^15^ it stretched into even the most arid districts of the Cape’s winter rainfall zone. It did not, however, reach the Cape Peninsula, allowing an initially tiny number of animals in the mid 17th century to propagate a viable colonial horse population.^16^ The first cases of horsesickness apparently occurred only during the late 17th century in the horses of hunters who entered zebra/quagga habitat in mountainous areas about 80 kilometres inland of Cape Town and in the plains to the north.^17^ By 1719, when the first recorded epizootic occurred, the colonial horse population, recorded at 2,548 animals in the previous year, was already sufficiently stable to absorb 1,600 to 1,700 horsesickness deaths, a mortality rate of about 65 per cent.^18^ Had it struck earlier in Cape Town, the VOC may have considered further imports of horses to be unjustified. Given the vital role of horses in exploration and conquest, any setback in the growth of the initial colonial horse population would have almost inevitably had a knock-on effect on colonial expansion at large.

In the mid 19th century, the whole of the country across the Vaal River and much of the Orange Free State was reportedly still affected by seasonally occurring horsesickness.^19^ The disease seems to have also been prevalent throughout Natal during the summer months; Wilhelm Bleek’s assertion that in Zululand horses were endangered even outside the horsesickness season in all probability points to the parallel prevalence of trypanosomiasis, but it could also indicate that this area used to be an endemic reservoir of horsesickness.^20^ Within the Cape Colony, most of the inland districts were at least partly affected by seasonal horsesickness. In the Western and Northern Cape this included large parts of the districts of Worcester, Clanwilliam, Beaufort and Colesberg. In the Eastern Cape, seasonal incidence of horsesickness was not only known in all the inland districts but could even occur in parts of the coastal districts such as Uitenhage and Albany.^21^

However, within the areas where horsesickness was seasonally prevalent, there were some, typically high-lying and often comparatively cold and/or dry, locations that were normally exempt or affected to a much lesser degree. Over time, local knowledge of these protected locations, and of the season during which the disease was prevalent, was acquired by farmers and indigenous horsemen through observation and sharing experiences orally.^22^ Indeed, even San raiders without horses of their own knew how to utilise horsesickness incidence to evade pursuit by mounted Boer commandoes.^23^ After the 1854–55 epizootic, the colonial government launched a comprehensive inquiry into the disease by sending out a questionnaire to all local officials. The replies give valuable insight into contemporary – and occasionally past – knowledge and understandings of the disease, even though it is generally not specified how and by whom this knowledge had been generated. This voluminous but otherwise unutilised data informed a contemporary pamphlet by gentleman farmer and racehorse breeder Thomas Bayley that was locally published.^24^

Seasonal removal of horses seems to have become a standard precaution against horsesickness losses in the interior of the Cape. John Barrow reported in 1806 that the top of the Hantam Mountain near the later town of Calvinia, an area where horsesickness was ‘endemic’, was a public pasture with ‘each inhabitant having the privilege of sending thither eight horses during the sickly season’.^25^ Knowledge of this mountain being exempt from horsesickness, and the practice of sharing its pasture between surrounding farmers, was first mentioned in 1787.^26^ Reserving high mountains as commonage for this purpose appears to have been an official VOC policy, which at least in the eastern part of the colony survived into the British period.^27^ After the British introduction of quitrents, claims to private ownership were laid upon some prime locations, for example, during the 1820s, the top of the Hantam.^28^ Nevertheless, some farmers with pastures free from seasonal disease continued to admit horses from less well-situated farms during the horsesickness season.^29^

Farms surrounding exempt locations like the Hantam became local foci of horse breeding. Another horse-breeding region in the Western Cape with high and cold pastures free of horsesickness was the Lower Roggeveld.^30^ In the Eastern Cape, horses were sent from the Camdeboo near Graaff-Reinet to the Sneeuberg by the 1770s.^31^ Yet, although some Sneeuberg farms were entirely free from seasonally occurring horsesickness, on others only six miles distant, it was almost impossible to keep horses alive.^32^ Conversely, outside these well-known regions, farmers would often know of small exempt places in their vicinity.^33^ Knowledge about horsesickness-free areas was constantly updated; for example, during the epizootic of 1854–55 it was observed that the Stormberg on the border of the Albert and the recently annexed Queenstown districts was also little affected.^34^ At the fringe of the area of seasonal horsesickness prevalence, exempt locations could even be situated at lower altitudes. For example, from the Langeberg north of Swellendam, farmers sent their horses to the Ruggens, a hilly plain to the south, during the horsesickness season.^35^

While this was a suitable measure for breeding horses and their offspring, some animals required for daily use had to be retained wherever the farmers were residing, even if thereby exposed to horsesickness. British military officer Colonel Richard Collins thus recommended that farmers in the Great Karoo should send most horses to the neighbouring mountains during the horsesickness season and keep ‘under cover at each place the few necessary to trace stolen cattle’.^36^ The general observation that stabling provided protection from the disease was common knowledge at least as early as 1775,^37^ but it is not clear when it was established that it was sufficient if done from well before dusk until a couple of hours after sunrise. Widely recommended as one of the principal precautions during the epizootic of 1838–39,^38^ overnight stabling avoided exposure at the time when *Culicoides* are most active. Nevertheless, it was primarily an option for sedentary horse-owners with animals valuable enough to justify the investment of building stables rather than coping with some losses to horsesickness, for example post contractors, racehorse owners and breeders of high-value animals.

There are no contemporary sources as to the morbidity and mortality in seasonally affected areas but estimates for late 19th-century Namibia, where horsesickness was then still largely endemic, may give a rough idea of the order of magnitude. It was estimated that over a period of ten years, the annual morbidity rate had averaged around 45 to 55 per cent, with a concomitant mortality rate of around 40 to 50 per cent for the whole horse population, reflecting the low survival rate in affected animals. The minimum overall mortality rate in the best of these ten years was estimated at about 15 to 20 per cent.^39^ While these figures cannot be directly transferred, it is not improbable that horse owners in seasonally affected areas of South Africa during the 18th and early 19th centuries had to accommodate annual horsesickness losses of perhaps ten to 20 per cent, even if they were able to take recourse to the few protective measures known.

### Horsesickness Epizootics

In those areas that are free from a seasonal prevalence of horsesickness, the disease occasionally breaks out in larger epizootics that include the whole region and entail very high losses because virtually all domestic equines are fully susceptible. In the mid 19th century, this dichotomy between seasonal, ‘endemic’ horsesickness and large-scale ‘epidemics’ was clearly perceived, and certain anomalies in the spatial pattern of seasonal incidence had been observed to foreshadow an imminent epizootic. Horse breeders in the coastal districts of the Western Province thus prepared for an epizootic when the disease appeared further west than Uitenhage.^40^ In Worcester, a district partly affected by horsesickness every year, an epizootic was expected when the disease approached from the direction of the eastern frontier,^41^ whereas during normal years it was seen to advance from the inland areas of seasonal prevalence to the north.

Table 1 lists years in which large epizootics have been recorded. It is often held that outbreaks take place at more or less regular intervals of ‘every 10 to 15 years on average’,^42^ or alternatively ‘roughly 20 to 30 years’,^43^ but the list reveals that there is in fact a high variance in these intervals. Furthermore, while such claims explicitly exclude the second half of the 20th century, when vaccination had greatly diminished the potential for vast epizootics, they fail to recognise that despite a sufficiently large horse population there were only three epizootics recorded during the entire 18th century. Of course, there is a possibility that additional years with severe mortality have not explicitly entered the historical record as epizootics. For instance, there were marked decreases in colonial horse numbers, by 21 per cent in 1736 and about 15 per cent annually in 1749 and 1750. As these were not reflected in corresponding dents in cattle and sheep numbers that could have indicated drought as a reason, and as they bypassed the districts situated immediately around the Cape Peninsula, they may well have arisen from horsesickness epizootics that have thus far eluded the historical record.^44^ Nevertheless, the markedly increased frequency of epizootics from the mid 19th century onward remains to be explained.

**Table 1.** Epizootics of African Horsesickness in South Africa. (Sources: Compiled from M. Baylis, P.S. Mellor and R. Meiswinkel, ‘Horse Sickness and ENSO in South Africa’, *Nature*, 397 (1999), p. 574; Coetzer and Erasmus, ‘African Horsesickness’, p. 460; M.W. Henning, *Animal Diseases in South Africa*, 2nd ed. (Johannesburg, Central News Agency, 1949), p. 582. The outbreak of 1763 is mentioned only in H. Watkins-Pitchford and A. Theiler, ‘Review: Investigations into the Nature and Cause of Horse Sickness’, *Agricultural Journal of the Cape of Good Hope*, 23, 2 (1903), p. 155, but seems to be confirmed by a 37 per cent drop in the colonial horse population: compare P. van Duin and R. Ross, *The Economy of the Cape Colony in the Eighteenth Century* (Leiden, Centre for the History of European Expansion, 1987), p. 151. According to contemporary sources, the outbreak listed in this literature only for 1839 was already very severe in Uitenhage and Albany during November and December 1838: Anon., [no title], *Graham’s Town Journal*, 29 November 1838, p. 3; Anon., 'The Horse Sickness: Extract of a Letter from Uitenhage, Dec. 10th, 1838’, *Graham's Town Journal*, 20 December 1838, p. 2. The evidence for possible epizootics in 1736 and 1749–50, not listed anywhere in this literature, is discussed below.)

	1801	
1719	1819	1913–14 1918
		1923 1925–26
1736?	1838–39	
1749–50?		1940 1946
	1854–55	1953
1763	1862–63	
	1877–78	
1780	1887–88	
	1891–92	1996

Table 2 lists the annual horse numbers that are available for much of the VOC period, and a quarter of the 19th century under British rule. These statistics are subject to many limitations in their enumeration, cover a variable, gradually increasing geographical area, and are greatly understated for the VOC period. For each of the periods covered by the two successive governments the level of underreporting should be largely consistent and the figures therefore give a useful indication of the general trend.^45^ The impacts of the horsesickness epizootics of 1719, 1763 and 1838–39 on colonial horse populations are clearly reflected in the figures. They far surpass the visible dents made by losses during the frontier wars of 1834–35 and, less marked, 1846–47, in magnitude. Well into the 19th century, horsesickness thus remained the single-most grave threat to horse-keeping in the colony.

**Table 2.** Number of Horses, Cape Colony 1653–1854. (Sources: Compiled from J. Burman, *To Horse and Away* (Cape Town, Human & Rousseau, 1993), pp. 17, 21–2, 27; S.S. Swart, *Riding High: Horses, Humans and History in South Africa* (Johannesburg, Wits University Press, 2010), pp. 21, 30–1; P. van Duin and R. Ross, *The Economy of the Cape Colony in the Eighteenth Century* (Leiden, Centre for the History of European Expansion, 1987), pp. 150–61; British Online Archives, *Cape of Good Hope Blue Book and Statistical Registers for 1828*, p. 308; *… 1829*, p. 342; *… 1830*, p. 388; *… 1831*, p. 402; *… 1833*, p. 317; *… 1834*, p. 324; *… 1835*, p. 254; *… 1836*, p. 292; *… 1837*, p. 254; *… 1838*, p. 244; *… 1839*, p. 250; *… 1840*, p. 282; *… 1841*, p. 276; *… 1842*, p. 280; *… 1843*, p. 281; *… 1844*, p. 324; *… 1845*, p. 310; *… 1846*, p. 382; *… 1847*, p. 382; *… 1848*, p. 420; *… 1849*, p. 450;; *… 1852*, p. 438; *… 1853*, p. 438; *… 1854*, p. 460., available at https://britishonlinearchives.com/collections/39/volumes/228/south-africa-cape-of-good-hope-1821-1909?filters%5bquery%5d=&filters%5bclassName%5d=document, retrieved 2 May 2025.)

Year	Horses	Year	Horses	Year	Horses	Year	Horses
1653	2	1725	2,069	1759	7,835	1793	15,435
1654	2	1726	2,176	1760	8,240	1794	No data
		1727	2,399	1761	7,298	1795	14,523
1660	43	1728	2,611	1762	8,457		
1662	40	1729	2,877	1763	5,329		
		1730	3,164	1764	6,077		
1681	197	1731	3,471	1765	6,207		
		1732	3,775	1766	5,487	1828	78,572
		1733	4,085	1767	7,006	1829	73,426
1700	928	1734	4,498	1768	7,004	1830	71,448
1701	681	1735	5,001	1769	7,437	1831	67,760
1702	746	1736	3,966	1770	7,883	1832	No data
1703	870	1737	4,271	1771	8,188	1833	78,159
1704	913	1738	4,430	1772	8,514	1834	67,987
1705	1,014	1739	4,728	1773	9,061	1835	64,042
1706	1,055	1740	5,142	1774	9,438	1836	63,301
1707	1,261	1741	5,193	1775	9,653	1837	79,881
1708	1,586	1742	5,623	1776	9,857	1838	71,793
1709	2,014	1743	5,789	1777	12,690	1839	56,703
1710	2,081	1744	5,749	1778	11,798	1840	62,986
1711	2,253	1745	6,193	1779	12,402	1841	64,104
1712	2,256	1746	6,597	1780	12,496	1842	74,594
1713	2,146	1747	6,776	1781	No data	1843	83,169
1714	2,176	1748	6,807	1782	11,891	1844	93,881
1715	2,290	1749	5,732	1783	12,384	1845	99,012
1716	2,325	1750	4,818	1784	13,114	1846	93,507
1717	2,356	1751	5,024	1785	12,386	1847	92,918
1718	2,548	1752	5,615	1786	12,521	1848	116,740
1719	1,586	1753	6,136	1787	15,314	1849	122,750
1720	1,143	1754	6,106	1788	13,358	1850	No data
1721	1,304	1755	6,852	1789	13,609	1851	No data
1722	1,428	1756	7,043	1790	14,438	1852	142,395
1723	1,753	1757	7,062	1791	14,088	1853	150,243
1724	1,881	1758	7,302	1792	14,343	1854	160,704

Nevertheless, the figures also reveal that after major horsesickness epizootics, the horse population recovered to its previous levels within less than a decade. The actual length of the recovery period varied and depended on the severity of an outbreak and the size of the population. After the epizootics of 1719 and 1762, when the total population was still below 10,000 animals, it took around eight to nine years to reach the pre-epizootic level. After the epizootic of 1838–39, a much larger population of around 80,000 animals was regained within four years. It has been estimated that the loss of 65,000 horses sustained during the epizootic of 1854–55 was similarly recovered by reproduction within just over half a decade.^46^

The causes of major epizootics have not yet been conclusively established, but most scientific explanations point to exceptional meteorological conditions that are particularly favourable for the vector. The size and spatial distribution of populations of *Culicoides imicola*, the principal horsesickness vector, increase with the duration of suitable climatic conditions, and virus replication escalates with the age of individual midges and the number of generations bred.^47^ The length of a particular *Culicoides* breeding season is therefore an important factor determining the extent and severity of epizootics. The epizootics of 1838–39 and 1854–55 were indeed characterised by an exceptionally long duration of horsesickness prevalence,^48^ and it appears as if all the major outbreaks during the second half of the 19th century began very early, in the last months of the year preceding the core horsesickness season in late summer around March.

It has been found that in the western part of South Africa, ‘where most of the major AHS epizootics have occurred’, there is ‘a very strong association between the timing of these epizootics and the warm (El Niño) phase of the El Niño/Southern Oscillation (ENSO)’; however, only certain El Niño years with a distinct rainfall pattern coincide with horsesickness outbreaks.^49^ These years are characterised by ‘pronounced drought in the early part of the ENSO year, followed by heavier rain than usual between April and June’.^50^ Only tentative explanations have been offered to account for this phenomenon, suggesting that either ‘[b]reeding sites of the vector may be altered or, during drought, the virus reservoir (zebra) may congregate near the few remaining sources of water where they are in contact with, and infect, more vectors. High temperature during drought increases vector population growth rates and favours virus transmission’.^51^ The subsequent rains then provide more breeding sites, resulting in the rapid dissemination of the vectors,^52^ the population growth of which is perhaps, as in locusts and mosquitoes, additionally boosted by their predators having been decimated during the preceding drought.^53^

However, this picture, derived from long-term and large-area data for the winter rainfall zone, contrasts with a ground-level observation from the eastern summer rainfall zone that ‘[e]arly and heavy rains followed by warm, dry spells favour the occurrence of epidemics’.^54^ In 1856, a landowner from the Swellendam area concluded from local inter-generational experience that horsesickness ‘has generally succeeded heavy rains, after long droughts, or great heat soon after the rains’, thus confirming both of the above alternatives without excluding either.^55^ At least for cases where El Niño events and associated horsesickness epizootics straddle two consecutive years, heavier than usual rain in the last quarter of the year, at the beginning of the horsesickness season, seems to be an important factor.^56^

Yet, while exceptional rainfalls seem to be a crucial determinant of epizootics, unbroken drought apparently did not prevent, and perhaps even increased, the regular seasonal incidence of horsesickness within the winter rainfall zone in the past. In December 1775, Anders Sparrman’s informants explicitly attributed an unusually early appearance of the disease in the Camdeboo district south of Graaff-Reinet to the severe drought that had prevailed in that year and persisted for at least a month after the outbreak.^57^ In 1805, horsesickness also appears to have broken out in the Roggeveld in the interior of the Western Cape during rather than after a drought.^58^ It seems possible that in these cases – neither coinciding with a large epizootic – both wild and domestic equines may have been compelled to use the same remaining water sources, thereby facilitating rapid virus transmission, because reservoir hosts and horses would have congregated in close proximity to each other, and to the few suitable sites for vector breeding, in temperatures that promoted both vector breeding and virus replication.^59^

In the 1854–55 epizootic, both meteorological constellations were observed: more rain than usual in the early part of summer in some of the Cape Colony’s eastern districts and the southern coastal strip of the Western Cape, but an extraordinarily dry spring followed by a very hot summer in the inland districts.^60^ This apparent contrast between climatic conditions in the seasonally affected area and the region hardest hit by the concurrent epizootic suggests that the ecology of horsesickness at the time may have been more complex than indicated by explanations centred largely on meteorological conditions favourable for the vector. Moreover, these climatic explanations do not seem to explain the relative rarity of recurring epizootics before 1780 and their increased frequency from around the mid 19th century.

It appears that the ecology of horsesickness had undergone some fundamental changes during the intermediate period that are not explained by meteorological peculiarities alone. In the following, it will be argued that sequential anthropogenic modifications of the environment were decisive for, first, the contraction of the area in which horsesickness was seasonally prevalent, and, second, the rise of frequent severe epizootics. During the earlier phase, the decisive change to the ecology of the disease appears to have been the hunting of wild equines to near-extinction, which reduced the reservoir host of the virus. During the later phase, more rapid horse movement, eventually in relays, seems to have allowed this fragile host to become an effective vehicle of its spread, while investment in artificial water sources additionally increased opportunities for vector breeding.

## Zebras, Quaggas and the Changing Ecology of Horsesickness

B.J.H. Barnard, a veterinary scientist at Onderstepoort Veterinary Institute, has suggested that horsesickness is explained in part by the size and distribution of zebra populations. The virus may overwinter in, and subsequently proliferate from, endemic foci with larger zebra populations. In those parts of South Africa where horsesickness occurs only seasonally or sporadically, herds are more isolated and generally below 100 individuals. Barnard asserts that after the first major outbreak in 1719, horsesickness:

continued to occur, on an almost country-wide basis, throughout the 19th century and for the first few decades of the 20th century, though the number of outbreaks declined throughout the period, particularly in the south of the country, [and that … t]his decrease coincided with the country-wide disappearance of large populations of zebra.^61^

Given the importance of the later process in this explanation, he is surprisingly vague in stating summarily that by the end of the 19th century zebras and quaggas had largely disappeared.^62^ To understand the role of wild equines in the changing ecology of horsesickness, a more precise periodisation of their reduction is required.

Assessments of the historical distribution of the three types of wild equines in South Africa that could serve as largely immune reservoir hosts for horsesickness virus – plains zebra, Cape mountain zebra and the now extinct quagga – are rendered difficult by the inconsistent and confusing use of names for them.^63^ The zebra referred to in historical accounts from the Cape would have in virtually all instances been the Cape mountain zebra. Even though a few historical accounts indicate the possibility of an occasional appearance of plains zebra just south of the Orange River, its occurrence there has never been adequately proved^64^ and it can thus be ruled out as a reservoir host for horsesickness in the Cape Colony.

The principal habitat of the Cape mountain zebra is rugged, mountainous territory. Its retreat into the most inaccessible parts of the Cape and its extreme wariness of humans observed during the second half of the 19th century may have been due to its decimation by hunting.^65^ In earlier times it was occasionally seen grazing in plains close to the mountains and some thought it may previously have been a plains animal which through successful adaptation to life in the mountains saved itself from extinction.^66^ But it is now clear ‘that their harder and faster-growing hooves inhibit the habitation of flat plains for long periods’,^67^ and they would have thus mainly left their mountainous habitats when necessitated by food pressure, for example during extreme winters.^68^ The mountain zebra was largely restricted to the mountain ranges that run parallel to the southern coast. In the Eastern Cape it occurred as far north as the Sneeuberg, but eastward its range extended apparently only as far as the Groowinterhoekberge, the Zuurberg and the mountains west of Somerset East and Cradock, respectively,^69^ thus roughly matching the border of the winter rainfall zone.

The question arises whether, like the plains zebra further north, populations of mountain zebra could have served as reservoir hosts capable of maintaining a continuous cycle of virus transmission that would have created local endemic foci for horsesickness. Present behavioural studies indicate that the size of stable nuclear breeding groups in mountain zebra is not much smaller than in plains zebra, usually comprising less than half a dozen individuals of reproductive age.^70^ But in their savanna habitats plains zebras often associated in large herds of 50 to more than 100 animals^71^ – the latter figure estimated by Barnard to be sufficient to sustain a continuous cycle of virus transmission. Quaggas were likewise known to gather in troops of 30 to 100 individuals.^72^ Given their harsh and remote habitats, it is likely that mountain zebra formed comparatively smaller herds that were relatively isolated from each other, except perhaps when moving into the plains during harsh winters when horsesickness was not prevalent. Such populations would have likely been too small to sustain the virus in a continuous cycle of transmission, and their typical habitat is also not very suitable for the principal vector of horsesickness. The mountain zebra is therefore unlikely to have been more than a secondary reservoir host for horsesickness.

It is thus likely that the quagga provided the principal reservoir for the horsesickness virus in the Cape. At one time, quaggas populated all suitable plains habitats within a range restricted by the Great Kei River to the east and the Vaal River to the north.^73^ Some uncertainty surrounds their disappearance in the wild. As late as 1843, quaggas were still found in large numbers on the northern and particularly the north-eastern plains of the colony, and they also still occurred in the Great Karoo. According to Henry Anderson Bryden, they became extinct in the Great Karoo by around 1858 and in the north-eastern plains, around Colesberg, very few were left by the 1860s.^74^ According to Emil Holub, a troop of about 50 was still seen in the Colesberg district in 1873.^75^ In the Eastern Cape, Thomas Baines still hunted quaggas along the Cacadou and Indwe Rivers, tributaries of the White Kei, in 1848.^76^ Even in 1856–57, some seem to have occurred near Cathcart on the border of Queenstown and British Kaffraria.^77^ Their extinction by hunting thus appears to have occurred gradually, with an expanding frontier of intensified pastoral production driving quagga and other wildlife before it, until the quagga eventually reached the limit of its suitable habitat somewhere close to the Vaal River.

It is evident that, at least in plains habitats of the Cape, wild equines had already been greatly reduced in numbers and distribution by the mid 19th century. According to Barnard’s hypothesis, the anthropogenic reduction of their habitat and population size during the first half of the 19th century and thereafter would entail a parallel decline in the frequency and severity of horsesickness outbreaks. Barnard does not state whether his claim of a progressive reduction in the ‘number of outbreaks’ refers to seasonal incidence of horsesickness or large epizootics, or both.^78^ If it includes seasonal incidence, it would be difficult to verify because there are no consistent long-term records. But if it refers to epizootics, it is clearly contradicted by historical evidence. Table 1 shows that, while from 1780 onward epizootics occurred roughly every other decade, their frequency actually increased after the 1838–39 outbreak, and during the second half of the 19th century there was an epizootic in every decade.

In terms of severity, the outbreaks of 1838–39 and 1854–55 each surpassed all previous epizootics. The latter is the most devastating on record, with an estimated 65,000 horses, about 40 per cent of the entire population, killed.^79^ It took place at a time when quaggas had already all but vanished from large parts of the Cape and particularly its southern coastal districts, the epicentre of this epizootic. The second-most severe epizootic, killing 25,000 horses, occurred as late as 1891–92,^80^ when the quagga was extinct, the mountain zebra on the verge of extinction and even the number of plains zebra further east had been greatly diminished.^81^ It is thus evident that the progressive disappearance of wild equines from the Cape did not result in a reduction of horsesickness incidence per se.

It does, however, appear that, parallel to epizootics becoming increasingly severe and frequent from around the second quarter of the 19th century, there was a gradual decline in the seasonal incidence of horsesickness. In 1830, Thomas Perry, then district surgeon of Graaff-Reinet, a district that previously had widespread annual horsesickness incidence, observed that after the epizootic of 1819 the disease had only reappeared in 1824, ‘but not to the same extent’, and then disappeared again until January 1829, when ‘it returned with a fatality equal to that of 1819’.^82^ A similar process seems to have occurred two decades earlier in the Overberg area between Caledon and Swellendam.^83^

In contrast, in those north-eastern districts of the Cape Colony and in the Great Karoo where quaggas still occurred during the 1840s and at least the early 1850s,^84^ namely Colesberg, Albert, Cradock and those parts of Beaufort and Worcester situated in the Karoo, horsesickness was still seasonally prevalent in the early 1850s.^85^ It also still occurred ‘more or less every year’ in the field-cornetcy of Camdeboo south-west of Graaff-Reinet,^86^ at the eastern extreme of the Great Karoo. But while it is not clear whether quaggas still survived here and in the adjacent, annually affected district of Somerset,^87^ the latter, together with Cradock to its north, did contain important refuges of mountain zebras.^88^ Beyond the Orange and particularly the Vaal River, where the process of hunting wild equines out of existence had progressed less far by the mid 19th century, horsesickness certainly remained an almost omnipresent seasonal disease.^89^

It thus appears that rather than precipitating epizootics, the presence of sizeable zebra and quagga populations was more decisive in delimitating the geographic area in which the disease occurred seasonally in every year. Today, the spatial extent of seasonal horsesickness incidence is primarily dependent on its dissemination from a few geographically restricted endemic foci by the vector, and thus delineated by climatic conditions facilitating its early and abundant breeding.^90^ In contrast, the seasonally affected area in the 19th century was much larger, suggesting that wild equines may have provided more, and geographically more widely distributed, endemic foci, as well as facilitated a more rapid and less climate-dependent seasonal dissemination of the virus than the vector alone would have been capable of.^91^ Nevertheless, while the progressive disappearance of wild equines can thus well account for the gradual decline of horsesickness as a seasonal disease, its evolution toward increasingly frequent and devastating epizootics remains to be explained.

## Infrastructure Development and the Rise of Epizootics

The gradual decimation of wild equines and other large wildlife was both a precondition and effect of the expansion and intensification of colonial settlement and pastoral production. This also involved a progressive increase of horse numbers, which eventually provided the virus with a host population large enough to allow its temporary reproduction and transmission independent of the wildlife reservoir. But because horses are accidental hosts and harbour the virus only for a brief period even if they are not swiftly killed, they could not sustain virus reproduction over extended periods, unless the virus was constantly transmitted to uninfected individuals within a very large area.^92^ Thus, for epizootics to occur with the increasing frequency and intensity observed during the second half of the 19th century, a change in epidemiological conditions must have allowed for horses to play a greater role in facilitating a significant spread of the virus beyond the shrinking region containing a more enduring reservoir of wild equines.

Regarding ecological changes affecting the vector of the disease, it has been noted that during the second half of the 19th century there was an increased incidence of a certain type of El Niño event correlated with horsesickness epizootics. This climatic pattern seems to have precipitated the breeding of exceptionally large *Culicoides* populations, resulting in a natural acceleration of virus distribution. But there also seem to have been other, anthropogenic factors promoting *Culicoides* breeding as well as rendering horses more effective in spreading the disease. These have been overlooked in scientific explanations of historical horsesickness epizootics.

In the arid environment of large areas of the Cape, rainfall was insufficient or too unreliable for large-scale cultivation, and even pastoral production often required transhumance to ensure that livestock had access to sufficient pasture and water. But with a rising population and a progressive stabilisation of settlement patterns, transhumance provided a solution only in sparsely populated inland areas, and even there trekking often took place between two or three fixed points.^93^ Thus sufficient water supplies for livestock and cultivation increasingly relied on investment in artificial water sources. In areas of intensive cultivation, irrigation trenches were already observed during the second half of the 18th century. But from the early 19th century, a real boom in the construction of dams – initially to catch water from springs but increasingly also to collect erratic rainwater – ensued on livestock farms. By the mid 19th century, artificial water supplies and irrigated gardens had become a common and widespread feature on farms throughout the colony.^94^ In British Kaffraria, large-scale government-funded irrigation projects were initiated within Governor George Grey’s ‘public works’ scheme from early 1855.^95^

These anthropogenic modifications of the environment not only facilitated an intensification of pastoral and arable production, but also provided ideal conditions for the principal horsesickness vector, *C. imicola*. In contrast to other, mostly dung-breeding *Culicoides* species, *C. imicola* breed in moist soil, and irrigation can therefore stimulate such prolific breeding that their numbers can increase 200-fold, resulting in their comprising above 99 per cent of the entire *Culicoides* population – a phenomenon that naturally occurs only in years with exceptional rainfall.^96^ Moreover, in arid areas, many breeding sites for these midges are created only through the establishment of watering points for livestock. Yet, while scientists have drawn a connection between recent increases in artificial watering and an expansion of the range and numbers of *C. imicola*,^97^ they seem unaware of how early it became widely utilised in the Cape and have thus not explored the possible link to the increased frequency of horsesickness epizootics from the mid 19th century onward.

Of course, many scientific and historical studies have identified strong links, albeit complicated by a wealth of concomitant socio-economic factors, between the creation of artificial water reservoirs and expanded breeding sites for mosquitoes, resulting in more widespread incidence or more perennial prevalence of malaria.^98^ However, the mosquito species serving as vectors for malaria breed directly in standing water, whereas *C. imicola* breed in moist soil. In arid areas without sufficient natural water supplies, mosquito breeding is thus more directly dependent on the dams themselves, and on practices like basin irrigation in rice cultivation,^99^ whereas *C. imicola* can already breed after the occasional soaking of soils by intermittent irrigation or even small spillages of water around troughs.

In malaria research, it has also been found that besides increasing the number of mosquitoes, dams and irrigation schemes often alter the species composition of local mosquito populations, even though it is site-dependent whether this results in an upsurge or reduction in disease incidence.^100^ In *Culicoides* populations, the breeding requirements of different species vary more fundamentally than between those mosquito species, but the vast alteration of species composition that commonly results from above-average rainfall – and likely from irrigation, too – generally favours *C. imicola*. It is thus quite probable that a more permanent provision of breeding sites through an increasing development of artificial water supplies during the early 19th century would have made the predominance of this species more perennial.

Increasing investments into the infrastructure of farms not only facilitated an intensification of arable and pastoral production that created ideal breeding conditions for *C. imicola*, but also opened previously unsuitable areas to more continuous grazing. Such spatial extension of husbandry and the general growth of livestock numbers would also have provided the two other *Culicoides* species known to transmit horsesickness with increased breeding opportunities over a wider area. Through their ability to breed in the dung of cattle, *C. bolitinos* and *C. gulbenkiani* can facilitate a penetration of horsesickness into areas where low temperatures or unsuitable soil composition prevent the breeding of *C. imicola*.^101^ Being able to breed in horse dung, the latter could even transmit the disease on pastures reserved for horses that were known as refuges from horsesickness. It appears that during exceptional outbreaks, these two species could indeed become significant vectors because while such locations were generally exempt from seasonal horsesickness, some of them were affected by the epizootic of 1854–55.^102^

By providing ideal conditions for the breeding of these three species of *Culicoides*, the intensification and spatial extension of pastoral and arable production provided horsesickness with a potential vector population that was more widely distributed and less rainfall-dependent than ever before. These three species are also possible vectors for bluetongue in sheep.^103^ Although temperatures suitable for breeding and virus replication still delimited the possible season, under these conditions the local presence or absence of the virus would have become the principal factor determining outbreaks of these diseases. In bluetongue, for which cattle serve as the reservoir, the perennial presence of a capable vector population may have often promoted an increased seasonal incidence rather than epizootics, and the disease seems to have indeed occurred seasonally in some areas where horsesickness had already disappeared as a seasonal disease.^104^ In horsesickness, which lacks such a livestock reservoir, the virus had to be reintroduced from its endemic foci into local horse populations in each new season.

After the disappearance of the wild equines that had previously either provided local reservoirs or facilitated a swift seasonal distribution of the horsesickness virus, its spread relied on short-lived vectors and fragile domestic hosts. Because *Culicoides* generally move only a few kilometres from their breeding sites,^105^ midges in adjoining populations only become infected gradually. Even where there are sufficient breeding habitats and susceptible hosts available within close distances, horsesickness will thus advance relatively slowly on a broad, relatively linear front through suitable habitats. This seems to be the predominant pattern of its seasonal spread in the region around its endemic foci in South Africa in recent decades. But the suitable season in the country would have rarely been long enough to allow for this mode of spread alone to carry the disease all the way into the south-western Cape, the epicentre of many epizootics, even if the range of wild equines extended much closer in the past. Besides, during epizootics, erratic outbreaks often occur at far distant places, indicating that the virus is sometimes more rapidly dispersed.

The most common route for the dissemination of the virus over long distances is the movement of infected hosts.^106^ While the transport of goods rested primarily on ox wagons, horses provided the principal means of personal transport throughout the colonial period. They were used daily to round up livestock, hunt, move on and between farms, and for traveling to nearby towns. The vital function of horses is illustrated by the fact that in 1854, the number of ‘work horses,’ which comprised largely of riding and draught horses, was at 56,893 almost exactly on par with the Cape Colony’s white male population of 56,407. As perhaps a quarter of this number would have been boys too young to ride, a considerable number of white women as well as Khoisan and African men would have also had access to horses, especially if allowing for some of the 103,811 horses that were enumerated as ‘breeding animals’ also being used for riding.^107^

Though trips on horseback were mostly local, extended journeys could cover a daily distance of around 60 miles.^108^ This daily average could be pushed up considerably; the *Albert Times* reported in 1857 that two men with three horses had covered 103 miles of rough road in 13 hours.^109^ From the second quarter of the 19th century, there was considerable investment in road infrastructure and horse wagons that could cover about 40 miles per day became common in private overland travel, especially in the more densely populated areas.^110^ Thus, by the mid 19th century, both the pace and volume of equine traffic on the Cape’s major routes had markedly increased.

This escalated movement of horses allowed for a swift transport of the virus along the colony’s roads, but its effective dissemination would have often been frustrated by the extreme vulnerability of horses to the disease. In many cases, infected horses that were ridden over extended distances would have died before passing the virus to uninfected midges during overnight rests, because the swift course of horsesickness is further accelerated by exercise, sometimes to a degree that death ensues without any prior symptoms.^111^ Although potentially rapid, the long-distance dissemination of horsesickness by conventional horse travel involved a lot of chance.

However, parallel to the increase in private horse travel, a public transport sector had also evolved out of the colonial postal system. By the mid 19th century, the mail on main routes was increasingly conveyed by privately operated horse-drawn post carts, which provided space for a few passengers. The post carts travelled non-stop at an average speed of seven to ten miles per hour, and horses were exchanged about every 20 miles.^112^ Contemporary advertisements for long-distance passenger travel illustrate the rapid speed with which the post carts traversed the colony. In 1857, the 240 miles from Burghersdorp to Grahamstown, for example, took less than three days, and the 326 miles from Burghersdorp to Port Elizabeth only three-and-a-half days.^113^ By the early 1850s, some of the colonial towns around Cape Town, Worcester and Port Elizabeth were even connected by regional omnibus lines which commonly tendered for the post on those routes. On peripheral routes, with less volume of mail and rudimentary roads, the post still conveyed on horseback, but here too, a relay system providing remounts to post riders facilitated rapid speed.^114^

This postal and public transport system raised the prospects of a successful transmission of the virus from travelling horses to local *Culicoides* and horse populations considerably, because horses were regularly exchanged at intermittent stages of the journey. Points of exchange were invariably situated at places with permanent natural or artificial sources of water, for the horses and travellers, and would have frequently harboured a *Culicoides* population capable of creating a ‘bridgehead’ for the virus. Within seven to ten days after feeding on a newly arrived infected horse, when the incubation period of these midges had passed,^115^ remount stations and roadhouses could thus provide a starting point for the spread of the virus through local *Culicoides* and horse populations, as well as for its further dissemination along the road.

Horses also remained essential for securing the highly volatile frontier region throughout the 19th century. For the military, larger numbers of horses were acquired only during actual war, but cavalry constituted the mainstay of the various militias and paramilitary police forces sequentially tasked with policing stock theft across the colonial border from the second quarter of the 19th century. At the same time, Africans in the Eastern Cape frontier zone also greatly increased their utilisation and breeding of horses, a trend reflected in the recorded thefts of 9,771 animals between 1810 and 1843.^116^ The unprecedented concentration of horses in this critical geographic zone may have created an additional host population for the transmission of horsesickness from its endemic reservoirs to the colonial farming districts and transport networks.^117^

It therefore seems evident that anthropogenic factors contributed considerably to the increased frequency and severity of horsesickness epizootics from the mid 19th century onward. While natural climatic patterns such as the ENSO cycle periodically boosted vector breeding, intensified and spatially expanded agricultural production in all probability provided for a permanently enlarged, more widely distributed and less rainfall-dependent *Culicoides* population comprised largely of capable vector species. Among an increasing horse population, these midges could facilitate massive outbreaks if infected with the virus. Although there is a natural seasonal spread of the virus through adjoining *Culicoides* and horse populations, it is likely that the escalating use of horses in long-distance travel and postal communication facilitated its vastly accelerated distribution over a larger area. Thus, while horses and midges alone could evidently not maintain the regular seasonal pattern of horsesickness incidence previously sustained in association with wild equines, they were capable of sparking and fuelling frequent large-scale epizootics.

## Conclusion

This article has demonstrated that the epidemiology of African horsesickness in the Cape was embedded in a complex interplay of socio-economic, ecological and biological factors. In no part of the Cape did horsesickness preclude frequent and widespread use of horses, despite annual losses of around ten to 20 per cent in areas with seasonal incidence. High death rates in the worst epizootics were debilitating but were recovered from within less than a decade. For much of the colony, however, the uncertainty about the survival of horses, and their cost, resulted in their minimal use for farm work such as ploughing, or the transportation of heavy goods. These tasks were performed by teams of oxen. In this indirect way, the disease helped the ox wagon to become more powerfully symbolic of settler South Africa than the horse, so celebrated in the colonisation of the American interior.

Nevertheless, for personal mobility and travel, the horse remained indispensable. Horses were also central to colonial military power and strategy and were widely adopted by African societies, particularly the Griqua, Sotho and Xhosa, where they were important in military resistance. Indeed, horses seem to have been most readily adopted by Africans inhabiting areas where horsesickness was not present.^118^ The impact of horsesickness on military campaigns is a complex area touched on in other work.^119^

The focus here is the marked transition evident in the epidemiology of horsesickness in the Cape from the late 18th century through to the 19th century. The decline of zebras and quaggas played some part, as did changing weather patterns. An intriguing additional possibility that could be investigated by future research in biomolecular archaeology would be the reconstruction of possible changes over the course of the colonial period in the African horsesickness virus itself, if viable remains can be recovered.^120^ But as has been demonstrated here, the rise of epizootics resulted largely from the unintentional side effects of anthropogenic modifications of the environment associated with the socio-economic development of the colony.

The epidemiological transition of horsesickness evolved in two phases that overlapped chronologically but were clearly distinct regionally. During the earlier phase, wild equines were exterminated regionally through intensive hunting as an integral part of an expanding pastoral economy. This progressively deprived the virus of its reservoir host, and the area in which horsesickness was seasonally prevalent contracted.

In the core settlement areas, where seasonal horsesickness generally disappeared first, and particularly in the coastal region of the Western Province, the cessation of annual losses to the disease reduced the cost and effort of keeping horses and thus made an intensification of their use in transport and farming more viable. Horse numbers grew in the first half of the 19th century, from about 15,000 in 1795 to around 150,000 in 1853, an increase perhaps only partly explained by the colony’s territorial and demographic growth. After the British occupation of the Cape, horses also assumed new and increasingly important roles in the colonial administration and economy. The swift postal system required for the effective governance of this large and sparsely populated colony could be facilitated only by horses. The requisite investment in road infrastructure allowed for increasingly fast overland travel by horse which was soon taken advantage of by private travellers and newly emerged public transport providers. These developments created unprecedented opportunities to derive substantial incomes directly from horses, which had previously rather been an indispensable utility than a profitable asset per se. However, most of the new horse-based ventures, such as post-conveyance tenders, public transport, and commercial horse-breeding propped up by the novel racing industry, required a considerable capital outlay and were therefore dominated by gentlemen farmers and wealthy entrepreneurs. Their influence on the government and public opinion through a sympathetic press and, from 1854, the colonial parliament, gave the lobby involved in the horse economy considerable weight.

The second phase of the epidemiological transition of horsesickness was in part a result of, but ironically also challenged, this newly gained economic and administrative importance of the horse. To a degree it also revived the potential of the disease to pose a strategic problem for frontier defence and policing. Large horsesickness epizootics, initially triggered by extraordinary climatic conditions facilitating a temporary explosion of vector populations, occurred about every 20 years from 1780 onward. While devastating throughout the colony, their impact was most severely felt in the core settlement areas where the progressive cessation of seasonal incidence had increased the utilisation of horses and detracted from previous precautions. These, however, remained sufficiently infrequent and restricted to allow recovery and then further growth of horse populations within a few years.

But by the mid 19th century, increasing investments into artificial water reservoirs and irrigation allowed for widespread vector breeding independent of rainfall, and henceforth sufficient temperature alone could trigger inflationary vector reproduction on an unprecedented scale. At the same time, the increased use of horses for travel and postal communication provided for a more rapid spread of the virus over a larger area, and even into locations from which the disease had disappeared. These mutually reinforcing processes facilitated large-scale virus transmission that was wholly independent of a reservoir of wild equines. The result was devastating epizootics that were restricted only by the length of the season suitable for vector and virus replication, or by the local availability of susceptible horses. These continued to occur almost every decade until horses were slowly replaced by mechanised transport, and science could finally provide a vaccine in 1934. The *Culicoides* midge was only identified as the most likely vector in 1944.^121^


Chris Andreas *Science Manager, Vechta Institute of Sustainability Transformation in Rural Areas, University of Vechta, Driverstrasse 22, 49377 Vechta, Germany. Email: chris.andreas@sant.oxon.org*
https://orcid.org/0000-0002-2140-1538


## Footnotes

1 P. Mitchell, *Horse Nations: The Worldwide Impact of the Horse on Indigenous Societies Post-1492* (Oxford, Oxford University Press, 2015), pp. 304–37.

2 Recent scientific literature often refers to the disease by its acronym, AHS. In retaining the customary name, this article follows other historical studies, in which the geographical descriptor ‘African’ is commonly omitted and horsesickness rendered in one word.

3 The reclassification of quaggas as subspecies of plains zebra rather than a separate species made it feasible to rebreed them: P. Heywood, *The Life, Extinction and Rebreeding of Quagga Zebras: Significance for Conservation* (Cambridge, Cambridge University Press, 2022).

4 D. Gilfoyle, ‘Veterinary Immunology as Colonial Science: Method and Quantification in the Investigation of Horsesickness in South Africa, c. 1905–1945’, *Journal of the History of Medicine and Allied Sciences*, 61, 1 (2006), p. 36.

5 J.A.W. Coetzer and B.J. Erasmus, ‘African Horsesickness’, in J.A.W. Coetzer, G.R. Thomson and R.C. Tustin (eds), *Infectious Diseases of Livestock: With Special Reference to Southern Africa*, Volume 1 (Cape Town, Oxford University Press, 1994), pp. 460–75.

6 B.J.H. Barnard, ‘Epidemiology of African Horse Sickness and the Role of the Zebra in South Africa’, *Archives of Virology Supplement*, 14 (1998), pp. 13–19.

7 J. Skotnes-Brown, *Segregated Species: Pests, Knowledge, and Boundaries in South Africa, 1910–1948* (Baltimore, Johns Hopkins University Press, 2024), pp. 67–104; K. Brown, *Mad Dogs and Meerkats: A History of Resurgent Rabies in Southern Africa* (Cape Town, University of Cape Town Press, 2011); P. Phoofolo, ‘Epidemics and Revolutions: The Rinderpest Epidemic in Late Nineteenth Century Southern Africa’, *Past and Present*, 138 (1993), pp. 112–43; P.F. Cranefield, *Science and Empire: East Coast Fever in Rhodesia and the Transvaal* (Cambridge, Cambridge University Press, 1991); C.R. Conz, ‘Sheep, Scab Mites, and Society: The Process and Politics of Veterinary Knowledge in Lesotho, Southern Africa, c. 1900–1933’, *Environment and History*, 26, 3 (2020), pp. 383–412; L. van Sittert, ‘Class and Canicide in Little Bess: The 1893 Port Elizabeth Rabies Epidemic’, *South African Historical Journal*, 48, 1 (2003), pp. 207–34.

8 W. Mwatwara, ‘Veterinary History in Southern Africa’, in T. Spear (ed.), *The Oxford Research Encyclopedia of African History* (Oxford, Oxford University Press, 2022), https://doi.org/10.1093/acrefore/9780190277734.013.1132; W. Beinart, *The Rise of Conservation in South Africa: Settlers, Livestock, and the Environment 1770–1950* (Oxford, Oxford University Press, 2003), pp. 128–57; D. Gilfoyle, ‘Veterinary Research and the African Rinderpest Epizootic: The Cape Colony, 1896–1898’, *Journal of Southern African Studies*, 29, 1 (2003), pp. 133–54; K. Brown, ‘Political Entomology: The Insectile Challenge to Agricultural Development in the Cape Colony, 1895–1910’, *Journal of Southern African Studies*, 29, 2 (2003), pp. 529–49; K. Brown, ‘Tropical Medicine and Animal Diseases: Onderstepoort and the Development of Veterinary Science in South Africa 1908–1950’, *Journal of Southern African Studies*, 31, 3 (2006), pp. 512–29; Gilfoyle, ‘Veterinary Immunology’, pp. 26–65; K. Brown, ‘From Ubombo to Mkhuzi: Disease, Colonial Science, and the Control of Nagana (Livestock Trypanosomosis) in Zululand, South Africa, c. 1894–1953’, *Journal of the History of Medicine and Allied Sciences*, 63, 3 (2008), pp. 285–322; W. Mwatwara, ‘‘‘The Tick Was Not Slow to Take Advantage”: Conflicts in the Struggle against East Coast Fever in Southern Rhodesia (1901–1920)’, *South African Historical Journal*, 65, 2 (2013), pp. 249–70; W. Mwatwara, ‘‘Even the Calves Must Dip’: East Coast Fever, Africans and the Imposition of Dipping Tanks in Southern Rhodesia, c.1902–1930’, *South African Historical Journal*, 66, 2 (2015), pp. 320–48; F. Dube, ‘‘‘In the Border Regions of the Territory of Rhodesia, There is the Greatest Scourge …”: The Border and East Coast Fever Control in Central Mozambique and Eastern Zimbabwe, 1901–1942’, *Journal of Southern African Studies*, 41, 2 (2015), pp. 219–35.

9 C.B. Andreas, ‘The Spread and Impact of the Lungsickness Epizootic of 1853–57 in the Cape Colony and the Xhosa Chiefdoms’, *South African Historical Journal*, 53, 1 (2005), pp. 50–72; C. Andreas, ‘Preventative Inoculation of Cattle against Lungsickness in the Cape: Informal Technology Transfer and Local Knowledge Production in the Nineteenth Century’, *South African Historical Journal*, 71, 4 (2019), pp. 536–59; W. Beinart, ‘Transhumance, Animal Diseases and Environment in the Cape, South Africa’, *South African Historical Journal*, 58, 1 (2007), pp. 17–41; W. Beinart and K. Brown, *African Local Knowledge and Livestock Health: Diseases and Treatments in South Africa* (Johannesburg, Wits University Press, 2013).

10 Gilfoyle, ‘Veterinary Immunology’, pp. 26–65; S. Vandenbergh, ‘“The Story of a Disease”: African Horsesickness and its Direct Influence on the Necessary Development of Veterinary Science in South Africa c. 1890s–1920s’, *Historia*, 55, 2 (2010), pp. 243–62; S.J.E. Vandenbergh, ‘The Story of a Disease: A Social History of African Horse Sickness, c. 1850–1920’ (MA thesis, University of Stellenbosch, 2009). For an exploration of earlier settler and state responses, see C. Andreas, ‘The Background to, and Impact and Management of, the Epizootics of Lungsickness and African Horsesickness in the Cape Colony, c. 1853–7’ (DPhil thesis, University of Oxford, 2017), pp. 140–77.

11 S.S. Swart, *Riding High: Horses, Humans and History in South Africa* (Johannesburg, Wits University Press, 2010); K. Brown, ‘Frontiers of Disease: Human Desire and Environmental Realities in the Rearing of Horses in Nineteenth and Twentieth-Century South Africa’, *African Historical Review*, 40, 1 (2008), pp. 30–57. For useful popular histories, see J. Burman, *To Horse and Away* (Cape Town, Human & Rousseau, 1993); D. Child, *Saga of the South African Horse* (Cape Town, Howard Timmins, 1967).

12 Coetzer and Erasmus, ‘African Horsesickness’, p. 462.

13 Coetzer and Erasmus, ‘African Horsesickness’, pp. 462–3.

14 P.S. Mellor and C. Hamblin, ‘African Horse Sickness’, *Veterinary Research*, 35, 4 (2004), p. 450: citing Barnard, ‘Epidemiology of African Horse Sickness’, who does not explicitly claim endemicity (compare pp. 13, 16) but indeed seems to imply it.

15 Swart, *Riding High*, p. 22.

16 Burman, *To Horse and Away*, pp. 16–21; Swart, *Riding High*, pp. 20–7.

17 Barnard, ‘Epidemiology of African Horse Sickness’, p. 16. Barnard writes ‘16th century’, but allusion to the subsequent epizootic of 1719 reveals this as a mistype.

18 P. van Duin and R. Ross, *The Economy of the Cape Colony in the Eighteenth Century* (Leiden, Centre for the History of European Expansion, 1987), p. 150; N. Penn, *The Forgotten Frontier: Colonist & Khoisan on the Cape’s Northern Frontier in the 18th Century* (Cape Town, Double Storey Books, 2005), p. 52.

19 W. Way, in T.B. Bayley, *Notes on the Horse-Sickness at the Cape of Good Hope, in 1854-’55: Compiled, by Permission of His Excellency the Governor, from Official Documents* (Cape Town, Saul Solomon, 1856), Appendix, p. 6.

20 W. Bleek, ‘Dr. Bleek’s Forschungen in Natal, 18. August 1855 Bis 18. Mai 1856’, *A. Petermanns Mitteilungen aus Justus Perthes Geographischer Anstalt*, 2 (1856), pp. 362–75.

21 Compare Bayley, *Notes on Horse-Sickness*, pp. 4, 6, Appendix, pp. 100–5.

22 This article follows Beinart and Brown’s lead of using the term local knowledge understood as capturing the sum of knowledge produced by settlers, various indigenous groups and locally based scientific experts, rather than imposing a conceptual boundary between indigenous and settler knowledge: Beinart and Brown, *African Local Knowledge*, 17–20.

23 J. Barrow, *Travels into the Interior of Southern Africa*, 2nd ed., Volume 1 (London, T. Cadell and W. Davies, 1806), p. 205. For additional examples, see Andreas, ‘Background, Impact and Management’, pp. 64–5.

24 Cape Archives Repository, CO (Colonial Office) 4502 (Papers re Horse Sickness 1855–1856); Bayley, *Notes on Horse-Sickness*. For a brief biography, see S. Dubow, *A Commonwealth of Knowledge: Science, Sensibility, and White South Africa, 1820–2000* (Oxford, Oxford University Press, 2006), pp. 79–80. For a detailed discussion of the development of contemporary local knowledge on, medical debates of, and practical measures against horsesickness, see Andreas, ‘Background, Impact and Management’, pp. 140–63.

25 Barrow, *Travels*, p. 355.

26 Penn, *Forgotten Frontier*, p. 85.

27 R. Hart, in T.B. Bayley, *Notes on the Horse-Sickness at the Cape of Good Hope, in 1854-’55: Compiled, by Permission of His Excellency the Governor, from Official Documents* (Cape Town, Saul Solomon, 1856), Appendix, pp. 87–8.

28 Nigel Penn (personal communication, 2008).

29 Hart, in Bayley, *Notes on Horse-Sickness*, Appendix, p. 87.

30 M.K.H. Lichtenstein, *Travels in Southern Africa, in the Years 1803, 1804, 1805 and 1806*, Volume 1 (London, Henry Colburn, 1812–15), p. 124.

31 M. Boucher and P.E. Raper (eds), *Robert Jacob Gordon: Cape Travels, 1777–1786*, Volume 1 (Houghton, Brenthurst Press, 1988), p. 83; compare T. Perry, ‘On the Horse Sickness’, *South African Quarterly Journal*, 1, 4 (1830), p. 442.

32 Barrow, *Travels*, p. 205.

33 A. Sparrman, *A Voyage to the Cape of Good Hope, Towards the Antarctic Polar Circle, and Round the World: But Chiefly into the Country of the Hottentots and Caffres, from the Year 1772, to 1776*, Volume 1 (Dublin, White, Cash, and Byrne, 1785), p. 237.

34 Way, in Bayley, *Notes on Horse-Sickness*, Appendix p. 7.

35 T. Moodie, in T.B. Bayley, *Notes on the Horse-Sickness at the Cape of Good Hope, in 1854–’55: Compiled, by Permission of His Excellency the Governor, from Official Documents* (Cape Town, Saul Solomon, 1856), Appendix, p. 60.

36 R. Collins, ‘Journal of a Tour to the North-Eastern Boundary, the Orange River, and the Storm Mountains, by Col. Collins in 1809’, in D. Moodie (ed.), *The Record: Or a Series of Official Papers Relative to the Condition and Treatment of the Native Tribes of South Africa* (Amsterdam, A.A. Balkema, 1960), part 5, p. 25.

37 Sparrman, *Voyage to the Cape of Good Hope*, Volume 1, p. 237.

38 H.E. Macartney, ‘[Letter] to the Editor’, *Graham’s Town Journal*, Grahamstown, 17 January 1839, p. 3; H. White, ‘Horse Sickness: [Letter] to the Editor’, *South African Commercial Advertiser*, Cape Town, 6 March 1839, p. 2; F.W.R. [Francis William Reitz, sr], ‘Horse Sickness’, *Cape of Good Hope Government Gazette*, Cape Town, 22 March 1839, pp. 6–7.

39 K.L. Sander, ‘Südafrikanische Epizootien mit besonderer Berücksichtigung der Pferdesterbe’, *Berliner Tierärztliche Wochenschrift*, 47 (1895), pp. 555–6.

40 Bayley, *Notes on Horse-Sickness*, pp. 4, 6.

41 Chief Commissioner of Worcester in T.B. Bayley, *Notes on the Horse-Sickness at the Cape of Good Hope, in 1854-’55: Compiled, by Permission of His Excellency the Governor, from Official Documents* (Cape Town, Saul Solomon, 1856), Appendix, p. 77.

42 Baylis *et al.*, ‘Horse Sickness and ENSO’, p. 574.

43 Coetzer and Erasmus, ‘African Horsesickness’, p. 460.

44 Compare van Duin and Ross, *Economy of the Cape Colony*, pp. 150–60.

45 Van Duin and Ross, *Economy of the Cape Colony*, p. 59; D. Nell, ‘‘‘For the Public Benefit”: Livestock Statistics and Expertise in the Late Nineteenth-Century Cape Colony, 1850–1900’, in S. Dubow (ed.), *Science and Society in Southern Africa* (Manchester, Manchester University Press, 2000), pp. 100–15.

46 Andreas, ‘Background, Impact and Management’, pp. 120–1.

47 R. Meiswinkel, E.M. Nevill and G.J. Venter, ‘Vectors: *Culicoides* Spp.’ in J.A.W. Coetzer, G.R. Thomson and R.C. Tustin (eds), *Infectious Diseases of Livestock: With Special Reference to Southern Africa*, Volume 1 (Cape Town, Oxford University Press, 1994), pp. 68–89; R. Meiswinkel and J.T. Paweska, ‘Evidence for a New Field *Culicoides* Vector of African Horse Sickness in South Africa’, *Preventive Veterinary Medicine*, 60, 3 (2003), pp. 243–53.

48 *Graham’s Town Journal*, 29 November 1838; Bayley, *Notes on Horse-Sickness*, Appendix, pp. 100–5.

49 Baylis *et al.*, ‘Horse Sickness and ENSO’, p. 574.

50 *Ibid*., p. 574.

51 *Ibid*., p. 574.

52 Mellor and Hamblin, ‘African Horse Sickness’, p. 454.

53 Beinart, *The Rise of Conservation in South Africa*, p. 42; M. Davis, *Late Victorian Holocausts: El Niño Famines and the Making of the Third World* (London, Verso, 2001), p. 49.

54 Coetzer and Erasmus, ‘African Horsesickness’, p. 462.

55 Moodie, in Bayley, *Notes on Horse-Sickness*, Appendix, p. 61.

56 Compare Baylis *et al*., ‘Horse Sickness and ENSO’, p. 574: Figure 1.

57 A. Sparrman, *Voyage to the Cape of Good Hope*, *Towards the Antarctic Polar Circle, and Round the World: But Chiefly into the Country of the Hottentots and Caffres, from the Year 1772, to 1776*, Volume 2 (Dublin, 1785), pp. 126, 173–4.

58 Penn, *Forgotten Frontier*, p. 262.

59 Mellor and Hamblin, ‘African Horse Sickness’, pp. 451–4.

60 Bayley, *Notes on Horse-Sickness*, p. 26.

61 Barnard, ‘Epidemiology of African Horse Sickness’, p. 16.

62 *Ibid*., p. 15.

63 Compare C.J. Skead, *Historical Mammal Incidence in the Cape Province 1: The Western and Northern Cape* (Cape Town, Department of Nature and Environmental Conservation of the Provincial Administration of the Cape of Good Hope, 1980), pp. 312–13, 328–30, 354.

64 *Ibid*., pp. 354–7; C.J. Skead, *Historical Mammal Incidence in the Cape Province 2: The Eastern Half of the Cape Province, Including the Ciskei, Transkei and East Griqualand* (Cape Town, Department of Nature and Environmental Conservation of the Provincial Administration of the Cape of Good Hope, 1987), pp. 257–8, 569–74.

65 H.A. Bryden (ed.), *Great and Small Game of Africa: An Account of the Distribution, Habits, and Natural History of the Sporting Mammals, with Personal Hunting Experiences* (London, Rowland Ward, 1899), pp. 93, 95–6.

66 Skead, *Historical Mammal Incidence 2*, pp. 574–5.

67 Y. Moodley and E.H. Harley, ‘Population Structuring in Mountain Zebras (Equus Zebra): The Molecular Consequences of Divergent Demographic Histories’, *Conservation Genetics*, 6, 6 (2005), p. 953.

68 Bryden, *Great and Small Game of Africa*, pp. 93–5.

69 Skead, *Historical Mammal Incidence 2*, pp. 575–6.

70 P.H. Lloyd and O.A.E. Rasa, ‘Status, Reproductive Success and Fitness in Cape Mountain Zebra (Equus Zebra Zebra)’, *Behavioral Ecology and Sociobiology*, 25, 6 (1989), pp. 411–20.

71 F.C. Selous, in H.A. Bryden, *Great and Small Game of Africa: An Account of the Distribution, Habits, and Natural History of the Sporting Mammals, with Personal Hunting Experiences* (London, Rowland Ward, 1899), p. 80.

72 Skead, *Historical Mammal Incidence 1*, pp. 339–40.

73 *Ibid*., p. 40; Bryden, *Great and Small Game of Africa*, pp. 77–8.

74 *Ibid*., p. 77.

75 E. Holub, *Seven Years in South Africa: Travels, Researches, and Hunting Adventures, between the Diamond Fields and the Zambesi (1872–79)* (Boston, Houghton Mifflin, 1881), p 38; compare Skead, *Historical Mammal Incidence 2*, pp. 565, 567.

76 R.F. Kennedy (ed.), *Journal of Residence in Africa 1842–1853 by Thomas Baines*, Volume 1 (Cape Town, Van Riebeeck Society, 1961), pp. 77–8.

77 Skead, *Historical Mammal Incidence 2*, pp. 294, 337, 350.

78 Barnard, ‘Epidemiology of African Horse Sickness’, p. 16.

79 Andreas, ‘Background, Impact and Management’, pp. 112–21.

80 R. Meiswinkel, ‘The 1996 Outbreak of African Horse Sickness in South Africa – the Entomological Perspective’, *Archives of Virology Supplement*, 14 (1998), pp. 69–83.

81 Barnard, ‘Epidemiology of African Horse Sickness’, p. 15.

82 Perry, ‘On the Horse Sickness’, pp. 438–9.

83 Moodie, in Bayley, *Notes on Horse-Sickness*, Appendix, p. 60; for a detailed case study, see Andreas, ‘Background, Impact and Management’, pp. 96–100.

84 Bryden, *Great and Small Game of Africa*, pp. 77.

85 Bayley, *Notes on Horse-Sickness*, p. 30; Appendix, pp. 100–3.

86 Field-cornet W.S. Ryneveld (Camdebo[o]), in T.B. Bayley, *Notes on the Horse-Sickness at the Cape of Good Hope, in 1854-’55: Compiled, by Permission of His Excellency the Governor, from Official Documents* (Cape Town, Saul Solomon, 1856), Appendix, p. 93.

87 Bayley, *Notes on Horse-Sickness*, p. 30.

88 Skead, *Historical Mammal Incidence 2*, pp. 575–6.

89 Way, in Bayley, *Notes on Horse-Sickness*, Appendix, p. 6.

90 Coetzer and Erasmus, ‘African Horsesickness’, p. 462.

91 See Andreas, ‘Background, Impact and Management’, pp. 27–32, for a detailed synthesis of the relevant scientific literature, and pp. 89–91 for a comparative discussion of the epidemiological effects of human–wildlife–vector interactions in horsesickness and trypanosomiasis.

92 Dogs are another accidental host of the virus, and it has therefore been speculated that they could be a relevant factor in its spread: S.J. Dennis, A.E. Meyers and P.J. Mitchell, ‘Diseases and the Distributions of Wild and Domestic Equids’, in H.H.T. Prins and I.J. Gordon (eds), *The Equids: A Suite of Splendid Species* (Cham, Springer, 2023), pp. 277, 280. However, this has been doubted as they usually seem to contract it from ingesting infected meat but are not readily preyed on by *Culicoides*: Coetzer and Erasmus, ‘African Horsesickness’, p. 463.

93 Beinart, *The Rise of Conservation*, pp. 39–40.

94 *Ibid*., pp. 89–93.

95 J. Lewis, ‘An Economic History of the Ciskei, 1848–1900’, Volume 1 (PhD thesis, University of Cape Town, 1984), pp. 218–9.

96 Meiswinkel and Paweska, ‘Evidence for a New Field *Culicoides* Vector’, p. 244.

97 Meiswinkel *et al*., ‘Vectors: *Culicoides*’, pp. 84–6.

98 R.M. Packard, ‘Agricultural Development, Migrant Labor and the Resurgence of Malaria in Swaziland’, *Social Science & Medicine*, 22, 8 (1986), pp. 861–7; J. Keiser, M. Caldas De Castro, M.F. Maltese, *et al*., ‘Effect of Irrigation and Large Dams on the Burden of Malaria on a Global and Regional Scale’, *American Journal of Tropical Medicine and Hygiene*, 72, 4 (2005), pp. 392–406.

99 W.R. Jobin, *Dams and Disease: Ecological Design and Health Impacts of Large Dams, Canals and Irrigation Systems* (London, E & FN Spon, 1999), pp. 64–5.

100 Keiser *et al*., ‘Effect of Irrigation ‘, p. 396.

101 Meiswinkel and Paweska, ‘Evidence for a New Field *Culicoides* Vector’, pp. 244, 250–2; Meiswinkel *et al*., ‘Vectors: *Culicoides*’, pp. 70: Table 5.1, 72: Table 5.2, 74, 82, 83: Table 5.5; J.T. Paweska, G.J. Venter and P.S. Mellor, ‘Vector Competence of South African *Culicoides* Species for Bluetongue Virus Serotype 1 (BTV-1) with Special Reference to the Effect of Temperature on the Rate of Virus Replication in C. *Imicola* and C. *Bolitinos*’, *Medical and Veterinary Entomology*, 16, 1 (2002), p. 10–20.

102 Andreas, ‘Background, Impact and Management’, pp. 110–11.

103 Meiswinkel *et al*., ‘Vectors: *Culicoides*’, p. 72: Table 5.2.

104 Moodie, in Bayley, *Notes on Horse-Sickness*, Appendix, p. 60.

105 Coetzer and Erasmus, ‘African Horsesickness’, p. 463.

106 *Ibid*., p. 463.

107 *Cape of Good Hope Blue Book and Statistical Registers for 1854*, pp. 346–7, 460.

108 Anon., ‘The Cape Horse as Adapted for Indian Cavalry’, *South African Agricultural News and Farmers’ Journal*, 1, 1 (5 July 1849), p. 5–7; Burman, *To Horse and Away*, p. 69.

109 Anon., ‘Local and Other News’, *Albert Times*, Burghersdorp, 7 November 1857, p. 2.

110 Burman, *To Horse and Away*, pp. 48–58.

111 Coetzer and Erasmus, ‘African Horsesickness’, p. 471; Mellor and Hamblin, ‘African Horse Sickness’, p. 449.

112 Burman, *To Horse and Away*, pp. 60–3, 68; Anon., ‘The Post-Cart’, *Cape Monthly Magazine*, 11 (1862), p. 22.

113 Anon., ‘Mail Cart’, *Albert Times*, 25 July 1857, p. 2.

114 Andreas, ‘Background, Impact and Management’, pp. 60–2.

115 Mellor and Hamblin, ‘African Horse Sickness’, p. 451.

116 Cape Archives Repository, BK (British Kaffraria) 113 (Kaffir Depredations 1850–1865), Statistics of the Eastern Border, No. 9: reproducing *Graham’s Town Journal*, 7 November 1839; Synopsis of the official returns … of depredations committed by [Xhosa], [Thembu], or [Mfengu] … 1838 to … 1843.

117 Andreas, ‘Background, Impact and Management’, pp. 56–9.

118 P. Mitchell, ‘The Horse in Southern Africa’, in T. Spear (ed.) *The Oxford Research Encyclopedia of African History* (Oxford, Oxford University Press, 2022), https://doi.org/10.1093/acrefore/9780190277734.013.580; Andreas, ‘Background, Impact and Management’, pp. 69–71.

119 Andreas, ‘Background, Impact and Management’, pp. 48–57.

120 The recent analysis of *Yersinia pestis* genome from the remains of a prehistoric Eurasian corpse indicates the potential of such research: J. Susat, H. Lübke, A. Immel, *et al*., ‘A 5,000-year-old hunter-gatherer already plagued by *Yersinia pestis*’, *Cell Reports*, 35, 13 (2021), 109278. Many thanks to the anonymous *JSAS* reviewer for this intriguing suggestion.

121 Gilfoyle, ‘Veterinary Immunology’, pp. 36, 59–60.

